# An Over Expression APP Model for Anti-Alzheimer Disease Drug Screening Created by Zinc Finger Nuclease Technology

**DOI:** 10.1371/journal.pone.0075493

**Published:** 2013-11-06

**Authors:** Xiaojing Zhang, Hui Li, Yiqing Mao, Zhixin Li, Rong Wang, Tingting Guo, Ling Jin, Rongjing Song, Wei Xu, Na Zhou, Yizhuang Zhang, Ruobi Hu, Xi Wang, Huakang Huang, Zhen Lei, Gang Niu, David M. Irwin, Huanran Tan

**Affiliations:** 1 Department of Pharmacology, Peking University, Health Science Center, Beijing, China; 2 Department of Laboratory Medicine and Pathobiology, University of Toronto, Toronto, Ontario, Canada; 3 Beijing N&N Genetech Company, Beijing, China; 4 Department of Pharmacology, Ningxia Medical University, Yinchuan, China; University of Lancaster, United Kingdom

## Abstract

Zinc Finger Nucleases (ZFNs), famous for their ability to precisely and efficiently modify specific genomic loci, have been employed in numerous transgenic model organism and cell constructions. Here we employ the ZFNs technology, with homologous recombination (HR), to construct sequence-specific Amyloid Precursor Protein (APP) knock-in cells. With the use of ZFNs, we established APP knock in cell lines with gene-modification efficiencies of about 7%. We electroporated DNA fragment containing the promoter and the protein coding regions of the zinc finger nucleases into cells, instead of the plasmids, to avoid problems associated with off target homologous recombination, and adopted a pair of mutated FokI cleavage domains to reduce the toxic effects of the ZFNs on cell growth. Since over-expression of APP, or a subdomain of it, might lead to an immediately lethal effect, we used the Cre-LoxP System to regulate APP expression. Our genetically transformed cell lines, w5c1 and s12c8, showed detectable APP and Amyloid β (Aβ) production. The Swedish double mutation in the APP coding sequence enhanced APP and Aβ abundance. What is more, the activity of the three key secretases in Aβ formation could be modulated, indicating that these transgenic cells have potential for drug screening to modify amyloid metabolism in cells. Our transformed cells could readily be propagated in culture and should provide an excellent experimental medium for elucidating aspects of the molecular pathogenesis of Alzheimer’s disease, especially those concerning the amyloidogenic pathways involving mutations in the APP coding sequence. The cellular models may also serve as a tool for deriving potentially useful therapeutic agents.

## Introduction

Alzheimer’s disease (AD) is a neurodegenerative disorder that causes progressive memory and cognitive decline during middle to late adult life. The AD brain is characterized by the deposition of amyloid β peptide (Aβ), which is produced from amyloid precursor protein (APP) by β- and γ-secretase (presenilin complex)-mediated sequential cleavage [1]. The fundamental hypothesis to describe the origin of AD is that Aβ initiates a toxic cascade that causes AD [2]. Adhering to the amyloid hypothesis, one can readily find a number of potential targets for disease treatment. As a result, considerable attention is being focused on developing therapies for AD that are directed toward metabolic pathways involving Aβ. Therapeutic interventions for AD have been developed over the past 20 years, though, treatment results remain unsatisfactory, and there have been few advances in new drug therapy or new directions in the treatment of AD. The reason for the lack of progress is in part due to the lack of a reliable preclinical research model.

Transgenic cells are desirable tools for new drug development as they provide a system with direct access to the cellular mechanisms that might suggest new potential drug targets. With the development of high-throughput screening (HTS) methods, the construction of suitable transgenic cell models should allow researchers to quickly conduct millions of chemical, genetic or pharmacological tests [3], and these tests should rapidly identify active compounds, antibodies or genes that would modulate a particular biomolecular pathway in AD pathogenesis. Cell lines derived from the human kidney or brain, primary neurons derived from mice and rats, or cells artificially over-expressing APP or presenilin with or without familial AD mutations have been utilized for in vitro studies [4,5]. These cell line models have proved to be great tools for drug screening, and permit the investigation of the cellular mechanisms of AD pathology. However, in these existing models, Aβ is either not stably expressed or is expressed at a low level, and it is often hard to identify off-target alterations [4–6]. What is more, owing to the low homologous recombination rate and the complexity of the process, it can take months to establish these cell lines [7]. Recently, the use of human induced pluripotent stem (iPS) cell-derived neurons for AD drug screening has been reported [8]. iPS cells provide a powerful new tool for the development of AD treatments since they have high Aβ production and react to typical inhibitors and modulators of the amyloidogenic pathway. Pluripotent stem cells, however, require even more complex procedures for their manipulation and considerable time for selection of the best clones [9]. In addition, iPS cells are not uniform even, with each clone being unique, thus limiting the ability to use iPS cells as a drug-screening model.

The recently developed engineered zinc finger nucleases (ZFNs), a chimeric fusion of a Cys_2_His_2_ zinc finger protein (ZFP) and the cleavage domain of FokI endonuclease, are considered to be reliable research and therapeutic tools for modifying specific genomic loci. Each Cys_2_His_2_ finger, the recognition motif of ZFNs, recognizes approximately 3 bp of DNA [10,11], thus three fingers of a ZFNs would bind a 9-bp target, enabling a ZFNs dimer (the active species) to specify an 18 bp DNA sequence as the cleavage site. Upon dimerization, the FokI domains, the cleavage motif of ZFNs, will cut DNA at the preselected site, introducing site-specific double-strand breaks (DSBs) into the targeted endogenous gene. Cellular DNA repair mechanism, induced by DSBs, increases the rate of HR by several orders of magnitude. ZFN-mediated gene modification has been successfully applied in rat [12], mouse [13], zebrafish [14–16], *C. elegans* [17], Drosophila [18], *Xenopus* oocytes [19] and *Arabidopsis thaliana* [20], achieving high efficiencies.

In this study, we successfully constructed APP over-expressing mouse fibroblasts cells using the ZFNs technology. Use of ZFNs results in high efficiencies of HR-mediated gene modification with a reduced spectrum of unwanted off-target alterations [6]. The cell lines we established express APP at a high level, and are capable of secreting Aβ into cell culture medium. Aβ42 production was inhibited in these cells by the α-secretase activator (donepezil), β-secretase inhibitor (galantamin) and by a nonsteroidal anti-inflammatory drug (NSAID, ibuprofen), suggesting that the expected amyloidogenic pathway produces it. The mutant APP knock in cell line, s12c8, presented greater susceptibility to drug treatment, compared to the wildtype APP knock in cell line w5c1. Transformed cells were readily propagated in culture and these cells should provide an experimental model to elucidate aspects of the molecular pathogenesis of AD, especially those concerning the amyloidogenic pathways involving mutations in the APP coding sequence and may also serve as a tool for deriving potentially useful therapeutic agents.

## Results

### *In vitro* assays of ZFN activity

**Figure 1:**
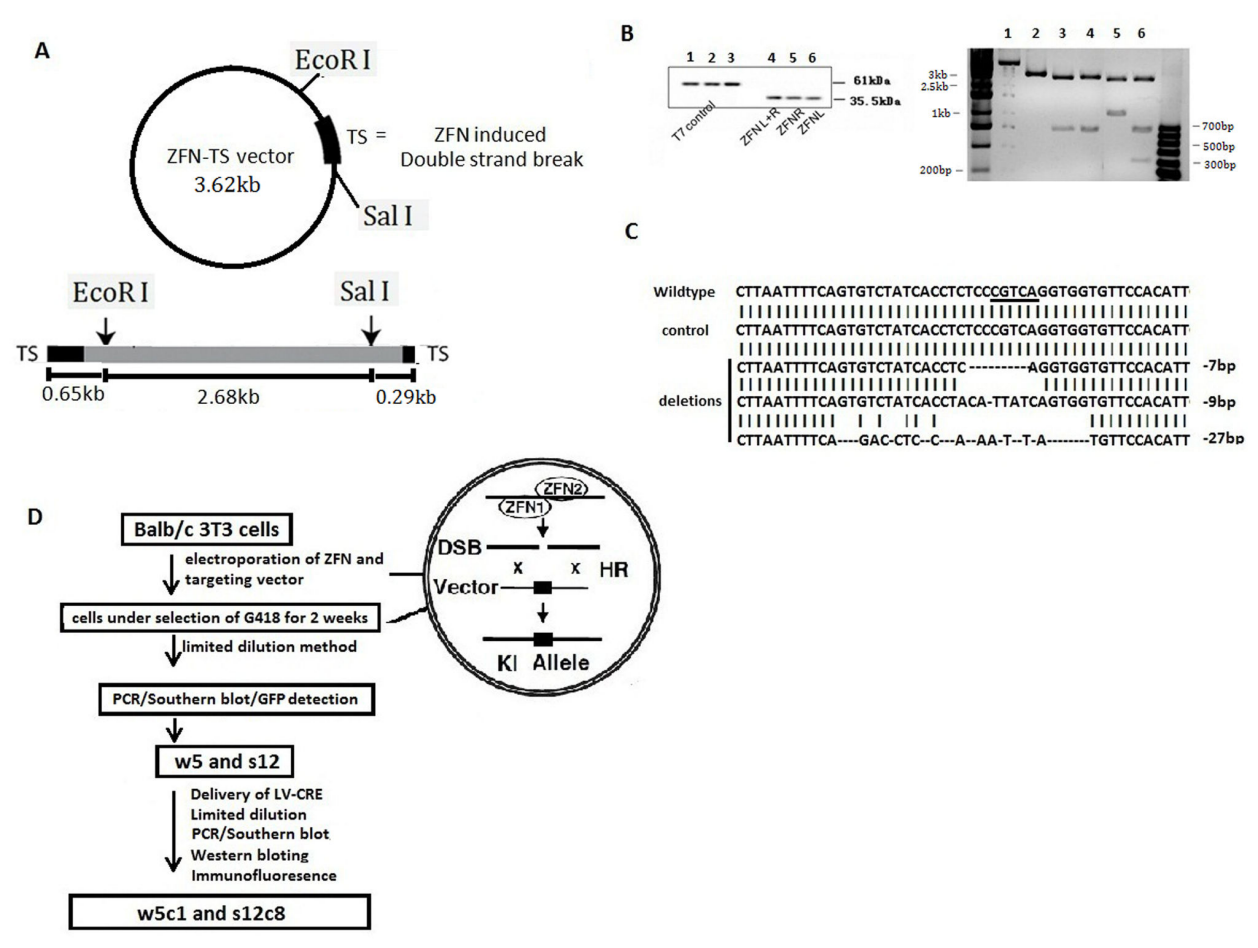
In vitro testing of the engineered ZFNs. (A) Plasmid ZFN-TS, for testing ZFN in vitro enzyme activity. DNA fragment corresponding to the ZFN target site (TS) was cloned into pMD-19T to form ZFN-TS. The expected sizes of the products generated by digestion of ZFN-TS by ZFNs and the restriction enzymes SalI and EcoRI are shown below the plasmid. (B) Identification of ZFN activity. ZFN proteins were generated using the IVTT reaction system and analyzed by SDS-PAGE electrophoresis (left). Lanes 1, 2, and 3 are T7 controls, which are translated into a 61 KDa protein - the expected size. Lanes 4, 5, and 6 are products of the ZFN left coding region (Lane 4), ZFN right coding region (Lane 5), and mixture of the ZFN left and right coding regions(Lane 6). ZFNs are translated as 35.5KDa peptides. Products of the digestion of ZFN-TS were identified by Agrose gel electrophoresis (right). Lane 1 is undigested ZFN-TS vector; lane 2 is digestion of ZFN-TS by EcoRI; lane 3 and lane 4 are double digestion by EcoRI and ZFN enzymes, using different orders of digestion (lane 3, EcoRI ZFN, lane 4, ZFN ◊EcoRI). Lane 5 is double digestion by EcoRI and SalI. Lane 6 is EcoRI, SalI and ZFN digestion. The sizes of the fragments are as expected. (C) Identification of ZFN activity in cells. ZFN activity produces heterogeneous mutations in the *Rosa26* *intron* *I* in cells. Sequence analysis was performed on 48 cloned mouse *Rosa26* alleles. The number of nucleotides deleted or inserted at the ZFN target site (underlined) in each clone is indicated to the right of each sequence. No mutations in the *Rosa26* were observed in a similar analysis performed with control samples from cells receiving empty vectors PMLM290 and PMLM292. (D) Schematic strategy for generating and identifying APP over-expression cell lines. Cells were electroporated with ZFNs and targeting plasmid and transformed cells were selected under selection with 600µg/ml G418 for two weeks. After limiting dilution methods, a total of 192 single-cell derived clones (96 mutant and 96 wildtype) were obtained and analyzed for integration into the ZFN-target site (spacer 5bp site in between the ZFN recognition sites in *Rosa26* *intron* *I*) by PCR, Southern blot analysis using the probe sp, and detection of green fluorescent protein (GFP) expression. Two clones, w5 and s12, were chosen as APP-inactivated cells (APP knock in cells but without APP expression). To activate APP expression, w5 and s12 were then transfected with LV-CRE to induce recombination and removal of the stop cassette. After limiting dilution, PCR and Southern Blot were used to test for deletion and APP activation at the DNA level. Western blot and immunofluorescence were used to test for APP and Aβ expression at the protein level. Subclones w5c1 and s12c8 were selected as APP overexpressing cells.

ZFNs were prepared using the TNT® Quick Coupled Transcription/Translation System (Promega). Translated crude proteins were incubated with the plasmid ZFN-TS (Figure 1A) which harbors a DNA segment that contains the ZFN targeting site within the *mouse Rosa26 intron I* to assess their DNA restriction activities in vitro. SDS-PAGE electrophoresis shows that the sizes of the ZFN proteins are 35.5KDa (Figure 1B) indicating that the ZFN plasmids were translated into the correctly sized proteins by the IVTT system. Digestion results of the ZFN-TS plasmid were analyzed using agarose gel electrophoresis (Figure 1B) where a single digestion of the ZFN-TS by EcoRI released a 3.62kb linear DNA fragment, which was then digested by the ZFN crude proteins to release two fragments of 0.65kb and 2.97kb indicating that the translated ZFNs cleaves the DNA at expected distance from the EcoRI site. To confirm the specific location of the ZFN cleavage site, the plasmid was digested by the ZFN as well as EcoRI and SalI that yielded fragments of 0.65kb, 0.29kb and 2.68kb in size (Figure 1B) consistent with the ZFN cleaving within the TS site. 

ZFN-induced double-stranded breaks repaired by nonhomologous end-joining (NHEJ) typically result in highly heterogeneous changes at the targeted locus [21]. We used this property to investigate whether Balb/c 3T3 cells transfected with ZFNs experience ZFN-induced NHEJ. A total of 48 individual *Rosa26 alleles* were sequenced after amplification from cells 48 hours after DNA fragments containing a promoter and ZFN coding sequences were delivery revealed that 3 alleles harbored mutations at the ZFN target site (Figure 1C). The mutated loci contained characteristic 7 to 27 bp deletions and the sequence diversity observed strongly suggested that the Balb/c 3T3 cells were modified by the ZFNs (Figure 1C). No mutations were detected from a control group (50 individual *Rosa26 alleles*), which were transfected with plasmids PMLM290 and PMLM292.

**Figure 2:**
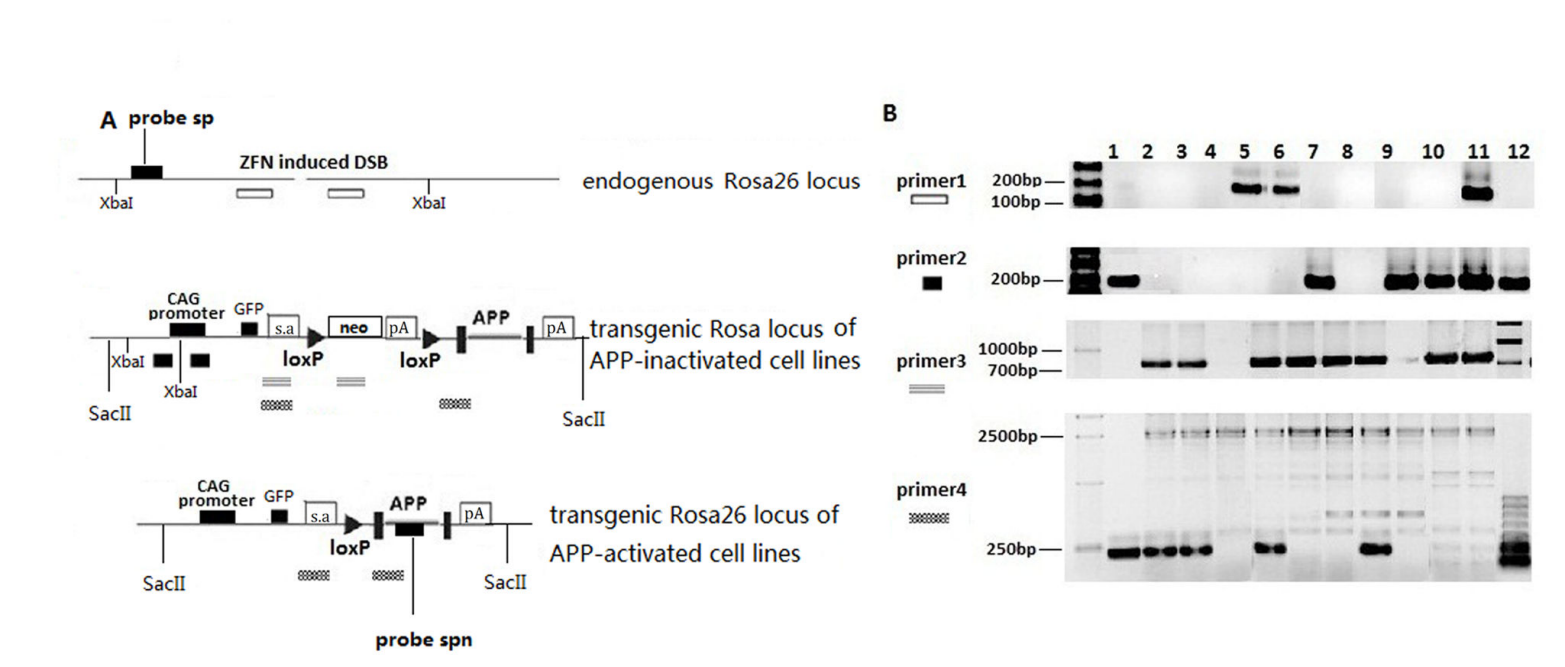
Identification of inactivated and activated APP expression cell lines using PCR. (A) Genomic locations of PCR primers and Southern blot probes for the inactivated and activated APP expression construct. Schematic illustration of the targeting constructs with locations of primers used to detect recombination events. To identify recombination events in the inactive APP expression cell lines, we use primer pairs primer1 and primer2. Cells with recombination events in both chromosomes will have PCR products of the following expected sizes: primer1: 0 bp; primer2: 215bp; In cells with a recombination event in only one of the two chromosomes then the PCR products will have the following expected sizes: primer1: 204 bp; primer2: 215bp; In cells with no recombination events the PCR products will have the expected sizes of: primer1: 204 bp; primer2: 0 bp; To detect deletion of the stop cassette in activated APP expression cell lines, we use primer pairsprimer3 and primer4. In cells with recombination events in both chromosomes then the PCR products will have the following expected sizes: primer3: 0 bp; primer4: 216bp; In cells with recombination events in only one of the two chromosomes then PCR products will have the following expected sizes: primer3: 801 bp; primer4: 216 and 2516 bp; In cells with no recombination events the PCR products will have the expected sizes: primer3: 801 bp; primer4: 2516 bp. (B) PCR analysis of inactivated/activated APP expression cell lines using primer pairs primer1, primer2, primer3 and primer4. Results using primer pairs primer1, primer2, primer3 and primer4 are shown. In the upper two panels (primer1 and primer2), lanes 1 to 5 are wildtype APP coding sequence knock-in cell lines and lanes 7 to 12 are mutant APP coding sequences knock-in cell lines. Lane 6 is PCR from untransfected Balb/c3T3 cell genomic DNA. In the lower two panels (primer3 and primer4), lanes 1 to 5 are wildtype APP coding sequence knock-in cell lines and lanes 8 to 11 are mutant APP coding sequences knock-in cell lines. Lanes 6 and 7 are PCR results of cell lines w5 and s12, respectively. For primer1 and primer2 recombination events were detected for both chromosomes in lanes 1, 7, 9, 10 and 12, which we named cell lines w5, s7, s9, s10 and s12. Cell lines w5 and s12 were then used for Cre transfection for removal of the stop cassette, as they the best GFP expression. Primer3 and primer4 were used to detect stop cassette deletion. Lane 1 shows that both stop cassettes were deleted from cell line w5c1. In lanes 2, 3, 5 and 8, stop cassette deletion was detected in only one of the two chromosomes, and these lines are named the cell lines w5c2, w5c3, w5c5 and s12c8. No stop cassette deletion was detected in negative controls w5 and s12 in lanes 6 and 7.

**Figure 3:**
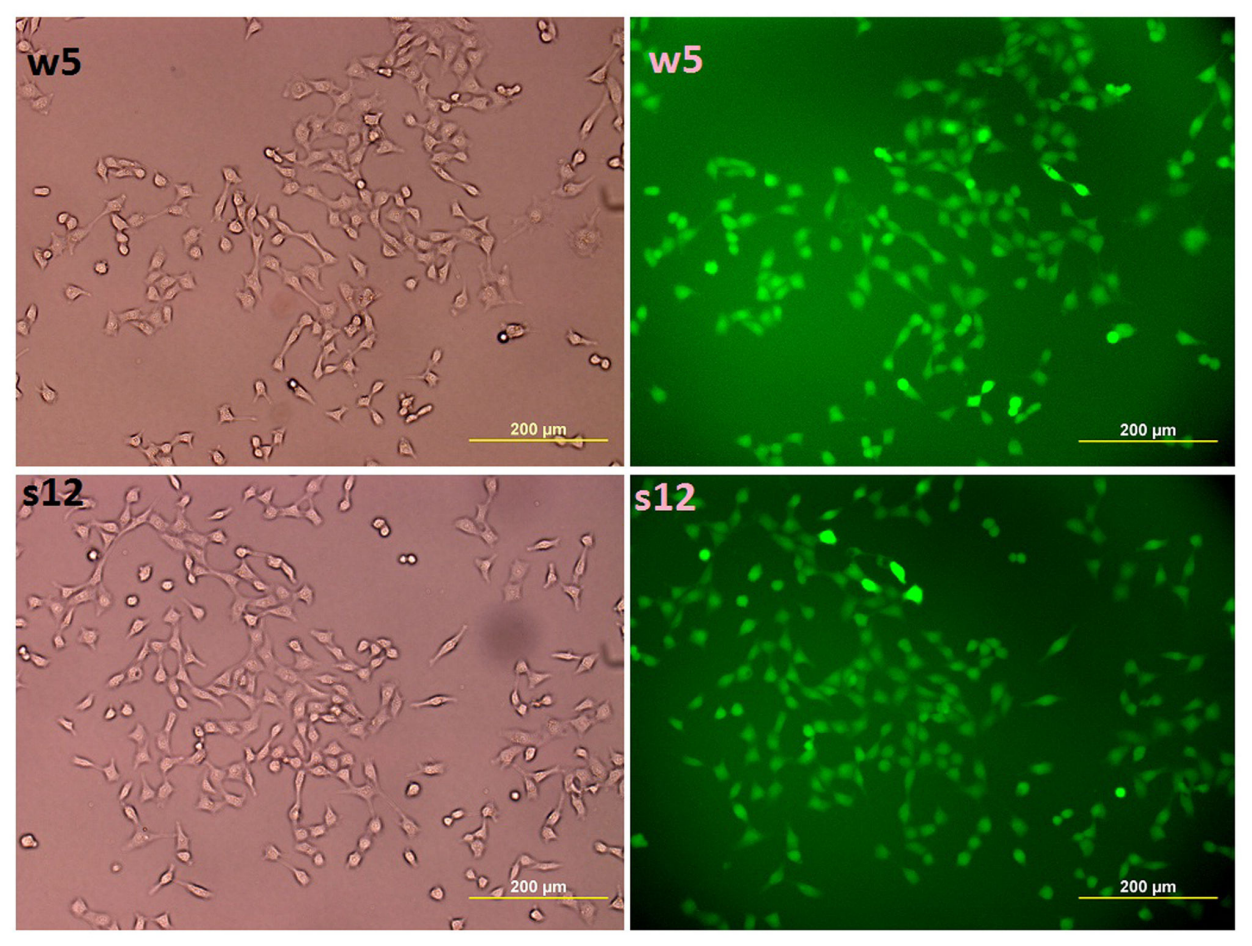
Expression of GFP Fluorescence in APP cell lines w5 and s12. Green fluorescence protein (GFP) was detected in cell lines w5 and s12, which do not have APP expression. GFP fluorescence expression was observed in more than 98% of the cells in both cell lines. Cells to the left were imaged under white light while those on the right under GFP excitation light.

Linearized DNA fragments containing the ZFN coding region and the CMV promoter along with the linearized APP targeting vector (Figure S1) were transfected into cells, which were then cultured in the presence of G418 (600µg/ml) for 2 weeks to select for transfomants (Figure 1D). A total of 192 single-cell derived clones (96 Swedish double mutation and 96 wildtype) were obtained by limiting dilution and analyzed for integration of the APP sequences into the ZFN-target site by PCR (Figure 2), Southern blot analysis using the probe sp (Figure 2), and detection of green fluorescent protein (GFP) expression (Figure 3). PCR analysis (Figure 2B) of the transformed cells (for the inactivated APP expression cassette) showed gene-modification efficiencies of 7.2 % (7/96, wildtype knock in) and 6.2% (6/96, Swedish double mutation APP knock in) (data not shown). Southern blot (Figure S2) analysis confirmed the PCR results. Cell lines s7, s9, s10, s12 and w5 (cell lines labeled with s contain the APP coding sequence with the Swedish mutation, while those with w are wildtype) were found to be transgenic at both chromosomes (i.e., integration of the APP cassette into both chromosomal alleles). GFP content, and localization, was directly detected by fluorescent microscopy, with results for cell lines w5 and s12 shown in Figure 3. Comparison of the fluorescence intensity revealed that cell lines w5 and s12 showed greater intensity than the other cell lines examined (results not shown).

### Transient Aβ over-expression induces apoptosis

**Figure 4:**
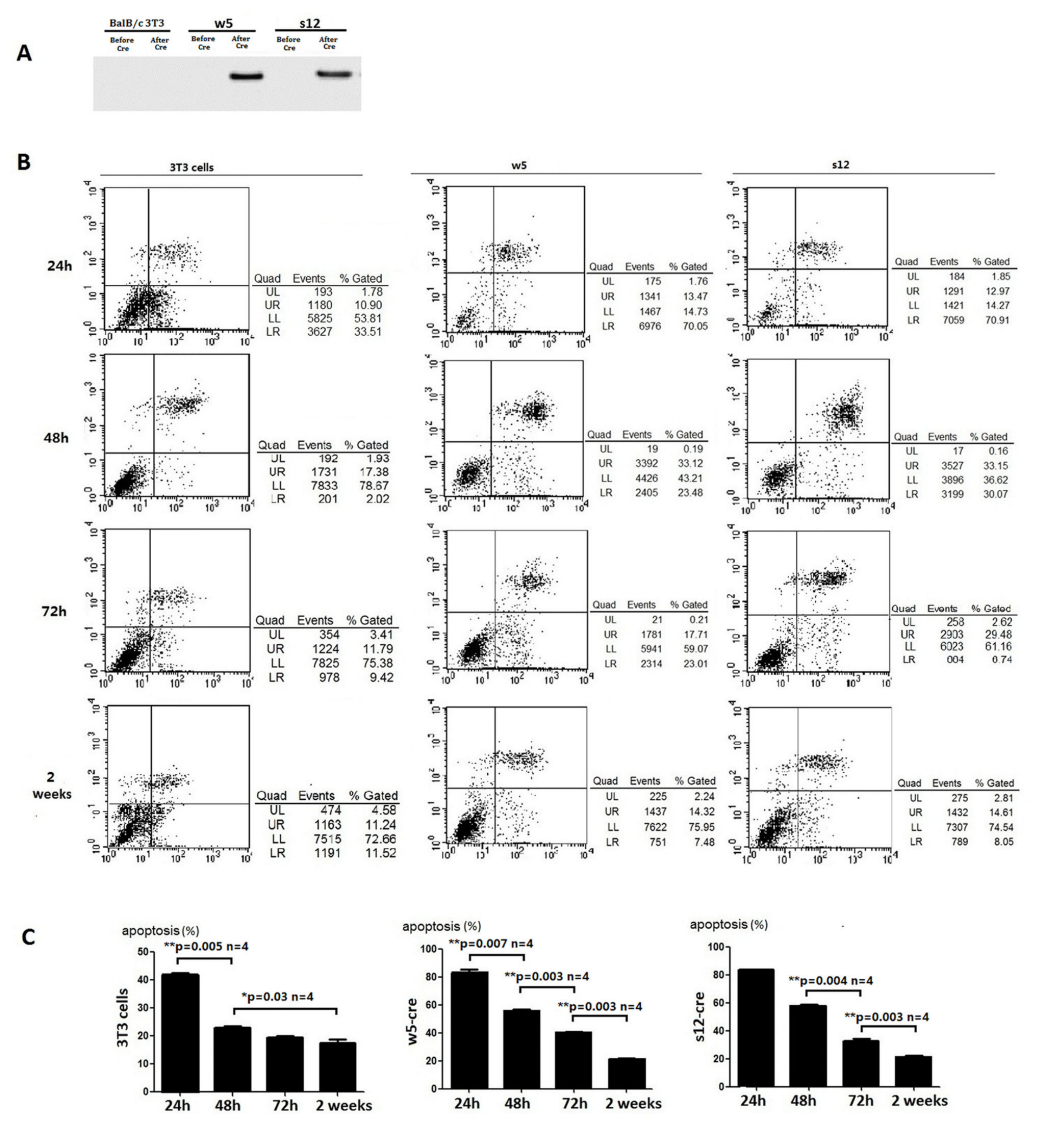
Analysis of apoptosis in transgenic cell lines after Cre induction. (A) Detection of Aβ expression in cell lines Balb/c 3T3, w5 and s12 before and after LV-CRE vector electroporation. Proteins in the cell culture medium were collected and equal amounts (100 μg/lane) were separated by SDS-polyacrylamide gel electrophoresis. Protein samples (s12 and w5) at different time points came from the same dish of cells. Media was collected every 24 hours and replaced with new media. (B) Change in the levels of apoptosis in the transgenic cell lines. Annexin V/Propidium Iodide staining and FACS analyses were used to quantify phosphatidylserine externalization and cell death in 3T3 cells, w5 and s12 after LV-CRE electroporation. The percentage of annexin V-positive cells is the sum of events in the upper right and lower right quadrants and the percentage of Propidium Iodide-positive cells is a sum of the events in the upper left and upper right quadrants. (C) Levels of apoptosis in the transgenic cell lines at different time points. Asterisk indicates statistically significant differences between two groups (*p<0.05, **p<0.01, ***p<0.001).

While the chicken gamma-actin promoter (CAG promoter) is located upstream of the APP cDNA, expression of APP is blocked by a STOP cassette located between the promoter and the APP coding sequence. Two LoxP sites flank the STOP cassette facilitating its removal and allowing induced APP expression. To remove the STOP cassette, and induce APP expression, we electroporated 10ug of LV-CRE (addgeneplasmid12105) vector into the cell lines w5 and s12 (which have the correct APP insertion), which results in the expression of CRE recombinase that removes the stop cassette located between the two LoxP sites. 24 hours after the LV-CRE vector was electroporated into the cells, Aβ expression could be detected (Figure 4A). At this same point in time, apoptosis was observed in the majority of the cells with both the w5 and s12 cell lines (Figure 4B). Apoptosis is in accord with previous reports that Aβ induces neuronal apoptosis [22], and might suggest that the w5 and s12 cell lines could be powerful tools for identifying drugs that protect cells from apoptosis due to β-Amyloid peptide over-expression. With subculturing, the apoptotic rate was found to decrease, where after 2 weeks the rate of apoptosis of the cells decreased to the baseline level (Figure 4C). Limiting dilution was then used to obtain stable single-cell lines that were activated to express APP. 

### Stable expression of APP and Aβ in the activated APP expression cell lines

**Figure 5:**
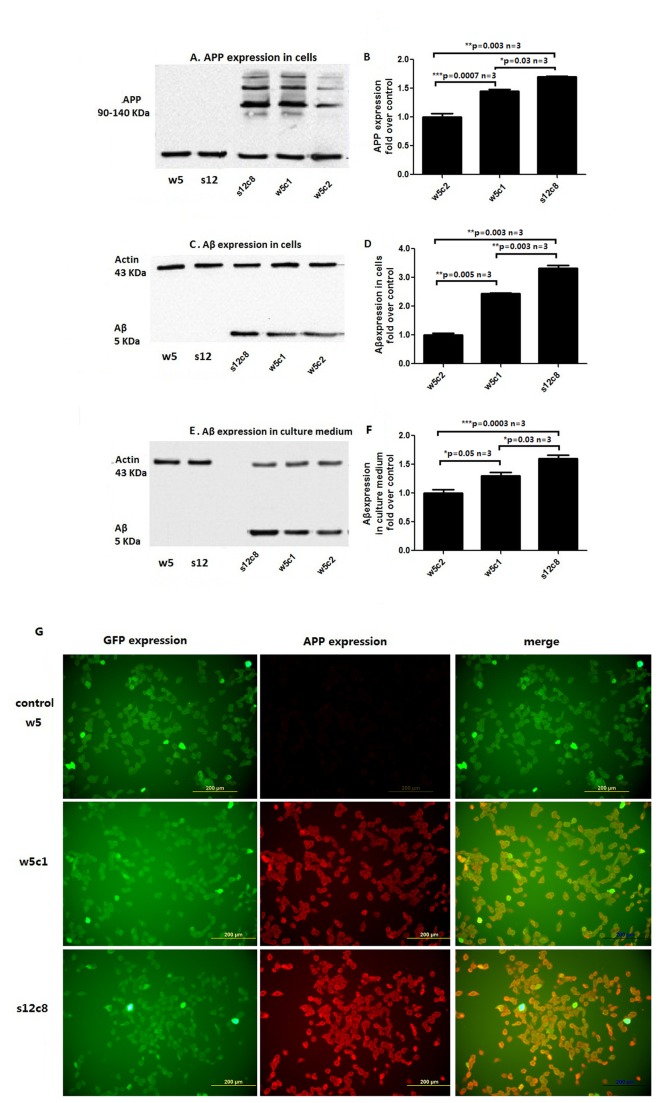
APP and Aβ expression in engineered cell lines. W5c1 and w5c2 are wildtype hAPP knock-in cell lines, while cell line s12c8 contains the hAPP sequence with the Swedish double mutation. (A) Top: Representative Western blot of homogenates of hAPP knock in cell lines using the human anti-APP Thr668 antibody (CST, #2452). Bottom: Representative Western blot for β-actin (CST, #4967) in the cell lines. APP expression was not detectable in cell lines w5 and s12. (B) Quantitation of the expression level of APP in cells. (C) Representative Western blot of cell homogenates from APP knock in cell line performed with mAb specifically recognizing Aβ isoforms (CST, #2454). (D) Quantitation of the expression level of Aβ in cells. (E) Representative Western blot of Aβ released into the cell media using mAb specifically recognizing Aβ isoforms (CST, #2454). While actin is found in the culture medium this likely is from dead cells. The ratio of Abeta/actin is higher in media (1.6:1) than in cells (1.1:1). This suggests that at least some of the Abeta were released from living cells. (F) Quantitative analysis of Aβ expression level in the cell culture medium. Results presented in B, D, and F were obtained from three independent experiments, each performed in triplicate and are expressed as mean ± SD. (G) Colocalization of GFP with APP using immunofluorescence. Green fluorescence protein (GFP, green) and Amyloid Precursor Protein (APP) immunoreactivity (red) co-localized in cell lines w5, w5c1 and s12c8. Cells from cell line w5 are positive for GFP but negative for APP. Scale bars=200µm. Asterisk indicates a statistically significant difference between two groups (*p<0.05, **p<0.01, ***p<0.001).

By limiting dilution, 60 single-cell derived APP clones (30 Swedish double mutant and 30 wildtype) were obtained and analyzed for stop cassette removal by PCR (Figure 2B) and Southern blot (Figure S2) analysis. Both methods confirmed the removal of the stop cassette in the w5c1, w5c2, w5c3, w5c5, and s12c8 cell lines. Western blot analysis was performed using anti-human APP Thr668 antibody (CST, #2452) and anti-human Aβ antibodies (CST, #2454). APP antibodies detected bands of the expected size of 90-140 kDa in cell lysates, and the Aβ antibody detected a band of the size 5kDa in both lysates and cell culture medium from cell lines w5c1, w5c2 and s12c8 (Figure 5A-F). While actin is found in the culture media, it is likely from dead cells. The ratio of Abeta/actin is higher in the media (1.6:1) than in cells (1.1:1) suggesting that at least some of the Abeta is released from living cells. Bands representing APP and Aβ were stronger in s12c8 than in w5c1 or w5c2 (Figure 5), indicating that the s12c8 line, harboring APP with the Swedish double mutation, produced more APP and Aβ than the w5c1 and w5c2 cell lines, which harbor the wildtype APP sequence. As both GFP and APP should be expressed from the transgene expression plasmid, we investigated the colocalization of GFP with APP in the cell lines w5c1 and s12c8 (Figure 5G). GFP-positive cellular structures appeared in green, due to GFP fluorescence, and APP-immunoreactive structures were in red, by TRITC, with the level of green fluorescence depending on the amount of GFP that has accumulated in the cells. Ectopic GFP expression was infrequent in APP-negative cells, and red-fluorescence was rarely seen in cells that lacked GFP-expression.

### Effect of ibuprofen, donepezil and galantamin on cellular APP and Aβ42 expression

**Figure 6:**
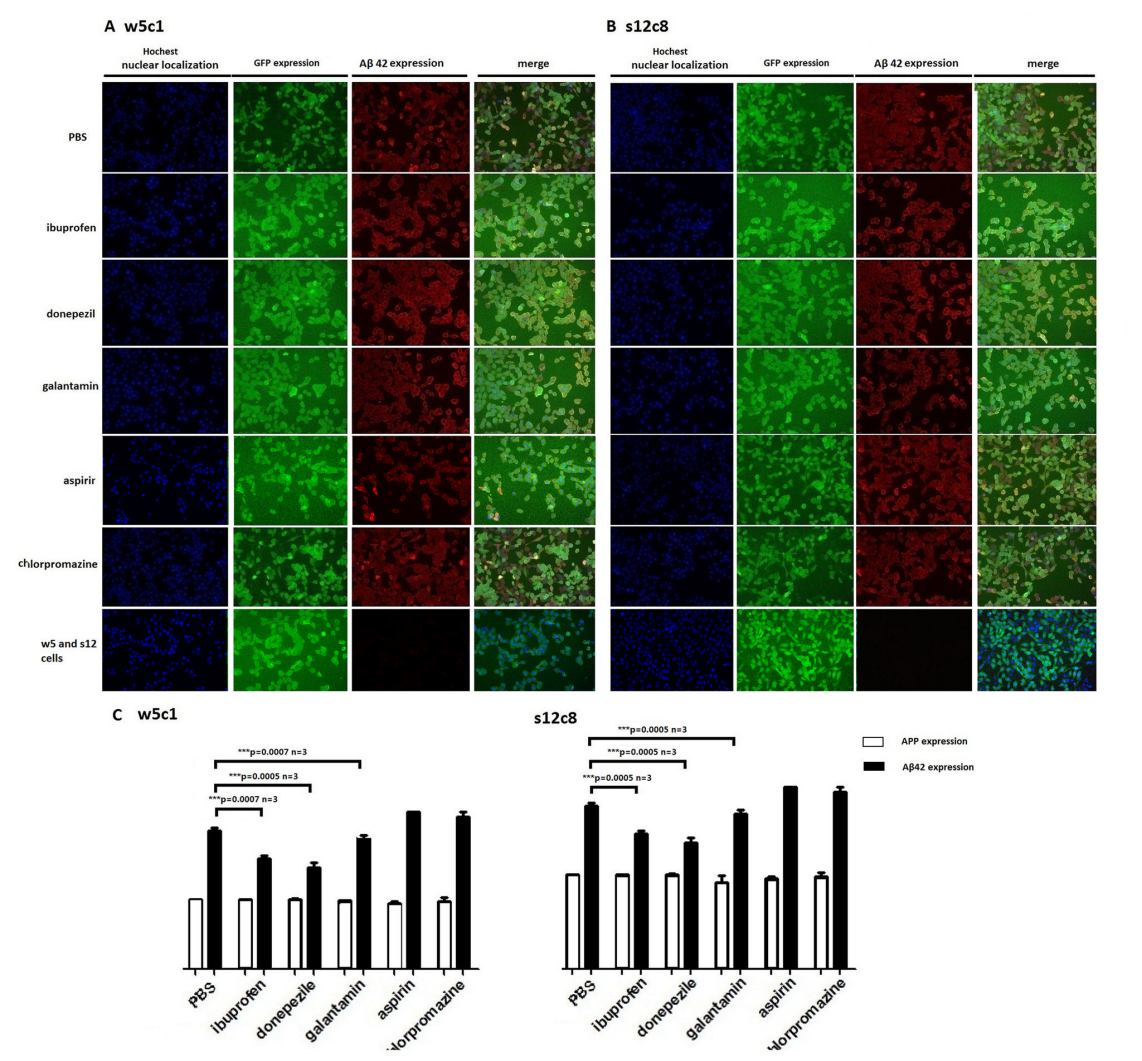
Effect of inhibitors of secretase activity on Aβ42 levels in our engineered cell lines assayed by High Content analysis. Cell lines w5c1 and s12c8 cells were stained for nuclei with Hoechst 33342 (blue), and GFP expression (green) was used to identify cells and APP location. Aβ42 expression was identified by tetramethylrhodamineisothiocyanate (TRITC, red). Images were acquired with the ArrayScan HCS reader with a 10x objective. Quantitation was performed with the Cell Health Profiling BioApplication (Cellomics, Pittsburgh, PA, USA). Cells were treated with ibuprofen, donepezil, galantamin, aspirin and chlorpromazine. PBS was used as a control. Data were obtained from three independent experiments, each performed in triplicate, and expressed as mean ± SD. Error bars represent SD. (A) Activity of drugs in cell line w5c1 measured by High Content. (B) Activity of drugs in cell line s12c8 measured by High Content. (C) Quantification of changes in APP and Aβ42 levels between drugs treatment groups. APP was detected in High Content using the anti-human APP Thr668 antibody as primary antibody, CST #2452, and a secondary antibody with TRITC as the reporter (data are not shown).Aβ42 expression in cells is significantly inhibited by ibuprofen, donepezil and galantamin in both the w5c1 and s12c8 cell lines. No significant change in the abundance of APP was seen in either cell lines after drug treatment. Results were obtained from three independent experiments, each performed in triplicate, and expressed as mean ± SD. Error bars represent SD. Asterisk indicates a statistically significant difference between two groups (*p<0.05, **p<0.01, ***p<0.001).

**Figure 7:**
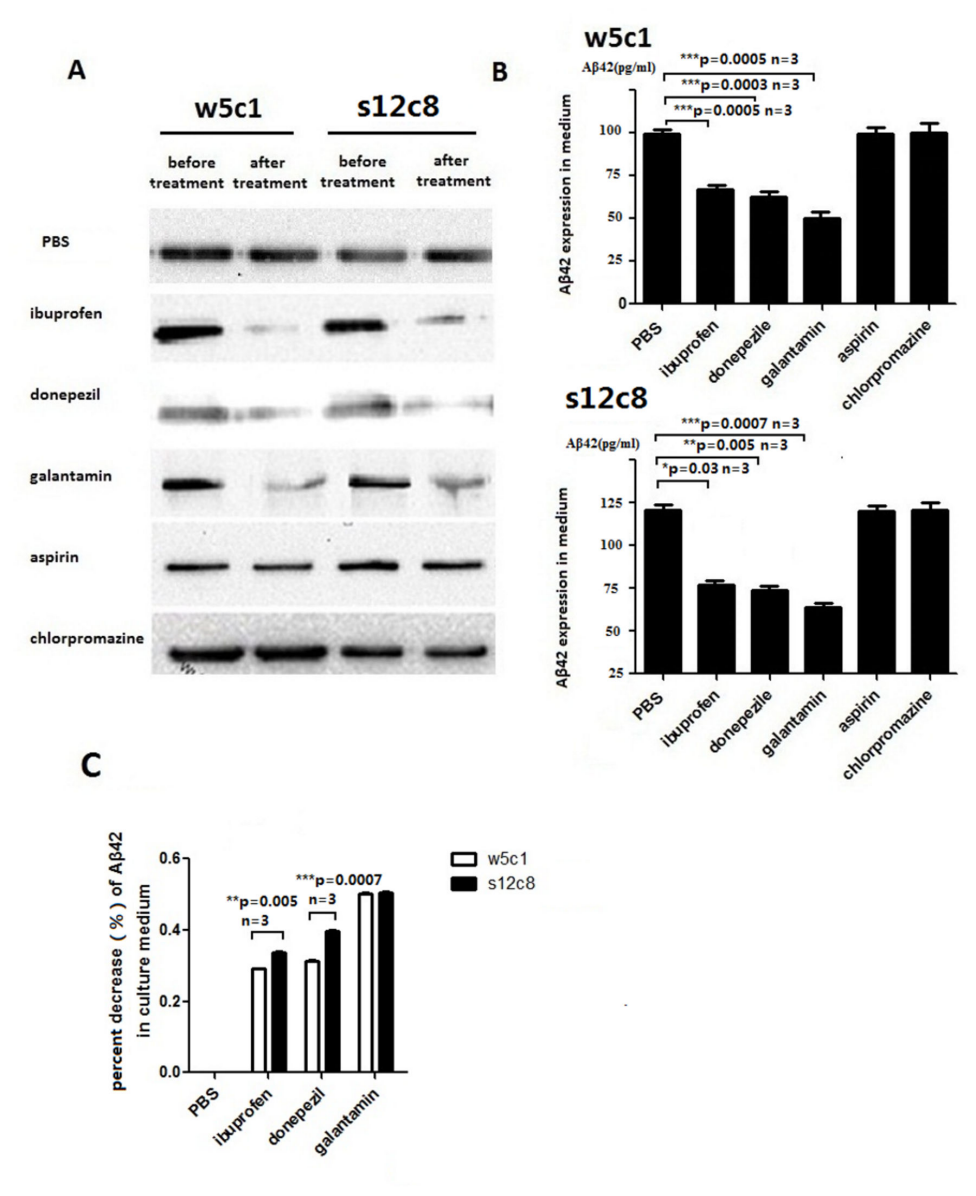
Changes in Aβ42 abundance in cell culture medium after drug treatment assayed by Western blot. (A) Western blot band intensity was used to measure differences in Aβ42 abundance in the cell culture medium of w5c1 and s12c8 cell after drug treatment. Representative blots are shown. Cells were treated with ibuprofen, donepezil, galantamin, aspirin and chlorpromazine. Results were obtained from three independent experiments, each performed in triplicate, and expressed as mean ± SEM. Error bars represent SE. (B) Quantification of the changes in Aβ42 levels between drug treatment groups. The release of Aβ42 into the cell culture medium was inhibited significantly by ibuprofen, donepezil and galantamin in both the w5c1 and s12c8 cell lines. Results are obtained from three independent experiments; each performed in triplicate, and expressed as mean ± SEM. Error bars represent SE. Asterisk indicates a statistically significant difference between two groups (P < 0.05). (C) Comparison of the effects of drugs on Aβ42 release into the cell culture medium by wildtype and mutant APP knock in cells. In ibuprofen and donepezil treatment groups, s12c8 exerted significantly greater sensitivity, compared to w5c1, to the drug treatments. Asterisk indicates a statistically significant difference between two groups (*p<0.05, **p<0.01, ***p<0.001).

To examine the effects of potential modulators of Aβ42peptide (the 42-residue isoform of the amyloid-β peptide) processing we used High Content (Figure 6) and Western blot (Figure 7) with anti-human APP Thr668 antibody (CST, #2452) and anti-Aβ42 antibody (CST, #7672) to identify changes in levels of the highly amyloidogenic Aβ42 peptide. We found that the NSAID ibuprofen treatment resulted in decreased levels of Aβ42peptide in our cell lines w5c1 and s12c8, a result similar to that seen by others in other cultured cells [23]. A decrease in Aβ42 levels was observed both within cells and in the culture medium upon ibuprofen, however, no significant change in the abundance of the holo-APP protein was observed (Figure 6C), indicating that the change in Aβ42 levels is due to changes in the rate of APP processing. In agreement with previous reports [24], aspirin treatment did not result in any significant change in Aβ42 isoform production or secretion, or APP levels (Figure 6C). The acetylcholinesterase inhibitors (AChEIs) donepezil and galantamin, which are used as the first line in pharmacotherapy for mild to moderate AD [25,26], exerted an Aβ42 lowering effect in the w5c1 and s12c8 transgenic cell lines, with significantly lower levels of Aβ42 detected both in cells and in the cell culture medium, with no change in APP levels, after treatment with these drugs (Figure 6 and 7). The antipsychotic drug chlorpromazine is effective for the treatment of schizophrenia [27] and was used as a negative control in our study. When our cells were treated with chlorpromazine no change in the abundance of APP or Aβ42 was observed either in cells or in the culture medium (Figure 6C and 7B). Quantification of the changes in the amount of Aβ42 secreted from cells into the culture medium revealed that cell line s12c8, which has the APP coding sequence containing the Swedish double mutation, was significantly more sensitive to drugs that inhibit Aβ42 production than the w5c1 cell line (Figure 7C; Table 1).

**Table 1:** Effects of inhibitors on the release of amyloid-β42 by wildtype and mutant APP knock in cells.

**Treatment**	**Aβ1-42 levels (pg/ml)**	**Decrease vs. PBS control (pg/ml)**	**Percent Decrease(%)**
**w5c1**			
PBS	99.32±0.49		
ibuprofen	70.65±0.62	28.59±0.26	28.9
donepezil	68.48±0.72	30.76±0.30	31.0
galantamin	49.20±0.62	50.04±0.15	50.4
aspirin	99.83±0.65	-0.59±0.18	
chlorpromazine	100.12±0.96	-0.88±0.47	
**s12c8**			
PBS	120.00±0.73		
ibuprofen	79.20±0.87	40.80±0.52	34.0
donepezil	72.20±0.81	47.80±0.35	39.8
galantamin	59.10±0.77	60.90±0.60	50.8
aspirin	119.80±0.73	0.20±0.47	
chlorpromazine	120.12±1.15	-0.12±0.79	

Cells were treated separately in medium with these inhibitors or PBS for 24 hours.

Each value shows the mean±SD of three cultures. N=3. ‘n’ represents the number of independent experiments. Statistical significance was determined by analysis of variance (ANOVA), followed by Tukey's post hoc comparison.

## Discussion

AD is the most common cause of dementia in the elderly and Aβ is thought to play a pivotal role in its pathogenesis [2]. Therapies for AD that are directed towards metabolic pathways involved Aβ formation have been developed for over the past 20 years, yet, results from these approaches have been disappointing [28–30]. Existing drug screening models are not satisfactory due to a number of problems including the complexity of procedures, low rates of HR rate, high off-target alterations, high cost and unstable Aβ expression [6]. We intended to construct a new drug screening cell model for AD, and focused the purpose of our study on two points: 1: Establishing gene knock in cell lines with a high gene modification efficiency and low unwanted off-target alteration rate.2: Constructing cell lines expressing APP at high levels that have reactivity to drugs that target key points in the amyloidogenic pathway or other pathways affecting APP metabolism. 

With the use of ZFNs, which have numerous applications in basic research, agriculture, and human therapeutics, we generated APP knock in cell lines with improved gene modification efficiency. APP knock in cell lines were established with gene-modification efficiencies of 7.2% (7/96, wildtype knock in) and 6.2% (6/96, mutant APP knock in), which are a bit lower than the efficiencies reported by others (as high as 10–20% [31–33]). Our rate of 7%, however, is much higher than the gene-modification efficiency generated by using traditional methods, which yield rates of approximately 0.01% [34], with a maximum reported frequency of about 0.4% [35]. The ZFN pair that we used targets an 18bp sequence ( cacctctcc **CGTCA** ggtggtgtt, with the target sequence underlined) that is located within the first intron of the *mouse Rosa26* gene. In target locus selection, three factors were considered: First, we need to select a site with high ZFP sequence-specificity and affinity. Second, since the affinity and sequence specificity of a designed ZFP for their target site is highest only when the individual ZF designs are chosen in the context of their neighboring fingers [36], we must consider compatibility between each chosen finger. Third, a suitable spacer between the two ZFNs recognition sites is strictly required. Using these criteria, we identified an 18bp sequence within intron 1 of the *Rosa26* locus as our ZFNs target site, and an 18bp sequence should be long enough to uniquely specify a single location within a mammalian genome [37–39].

To increase gene modification efficiency, and avoid unnecessary off target alterations, we adopted the mRNA transference method, where a DNA fragment containing a promoter and the protein coding region of interest are electroporated into cells, to generate mRNAs for a heterodimeric FokI based ZFN nuclease. Currently, the two preferred methods to express ZFNs are: (1) transfection of ZFN-expression plasmids [6,37] or (2) transfection of linear DNA fragments encoding ZFN mRNAs [40,41]. While further study is necessary to determine which of these methods is most suitable for modifying genes using ZFNs, current knowledge indicates that there are distinct differences between these two approaches. Transfection of plasmids can give rise to the possibility of permanent expression of the ZFNs as a result of the integration of the plasmid into the host genome [42,43], and this integration increases the risk of off-target effects due to the possibility that the ZFN expression construct being integrated into an endogenous gene and thus inducing an undesired insertional mutation. Transfection of a ZFN-encoding linear DNA fragment generally avoids problems associated with insertional mutagenesis, but typically has more transient expression of the ZFN. In the present study, we exploited the advantages of the mRNA transference method, after our in vitro assays proved the efficiencies of the ZFNs (Figure 1). DNA fragments that included the ZFN coding region and the CMV promoter were introduced by electroporation into mouse Balb/c 3T3 fetal fibroblasts, using an approach to minimize ZFN-mediated mutagenesis induced cytotoxic effects on cell growth by using a pair of mutant FokI mRNA. Since toxicity in mammalian cells is often due to cleavage of non-target sequences [44], and off-target breaks by ZFNs are largely caused by the activity of ZFN monomers that form homodimers that target incorrect sites, these effects can be significantly reduced by using FokI nuclease variants that can only form heterodimers [44]. In summary, the use of an approach with a pair of mutant ZFNs allowed us to construct a gene knock in cell model with a high HR efficiency and with a low rate of off-target alterations, and illustrates that this approach can also potentially also be used by other investigation into protein function.

The Cre-LoxP system facilitated our studies as we could generate cell lines that do or do not express APP, depending upon their exposure to Cre. Over-expression of APP, or a subdomain of it, may lead to an immediately lethal effect, thus preventing the use of transfected cells for further investigations. Indeed, we observed apoptotic rates of over 80% 24 hours after LV-CRE (plasmid containing Cre coding sequence) delivery into the cell lines w5 and s12, where Cre expression activates APP expression (Figure 4). Aβ was detectable in the cell culture medium from these cell lines treated with Cre(Figure 4A), suggesting that increased levels of APP or Aβ might have induced cell death. The high apoptotic rates observed in these cell lines could be a useful feature, as LV-CRE transfected w5 and s12 could be used to screen for drugs that protect these cells from the harmful effects of the Aβ isoforms in vitro. Despite the potential deleterious effects of amyloid over-production, studies of the essential metabolic processes in APP expressing cells now appear accessible. Some cells that over-express APP were found to survive through transfer of culture, leading to our development of the cell lines w5c1 and s12c8. As an expression cassette for Green fluorescence protein (GFP) was inserted upstream of the APP coding region in our HR template, and yields a GFP-APP fusion protein, we confirmed that cells in both of our cell lines expressed GFP and were positive for APP, thus we could use GFP to identify cells that express APP (Figure 5).

Both the wildtype and Swedish double mutation APP coding sequences are both in expression constructs that use the same Chicken gamma-Actin (CAG) promoter, with a CMV enhancer, followed by an expression cassette for Green fluorescence protein, and suppressor sequences, and both are inserted into the same genomic locus, the *Rosa26* intron sequence. The similarity of the genetic control region for both APP constructs (wildtype and Swedish double mutation) should yield parallel patterns expression for both constructs. The cell line w5c1 has two copies of the wildtype APP sequence, with one inserted into each of the two *Rosa26 alleles* in the genome of this cell line. In contrast, the cell lines 12c8 has only a single copy of the APP sequence (the Swedish double mutation), as only one of the two *Rosa26 alleles* has an inserted transgene. When we characterized the w5c1 and s12c8 cell lines in terms of expression of APP and Aβ, both produce immunologically detectable APP and Aβ (Figure 5). Despite the similarity in genetic control, and having only one copy of the expression construct, the levels of APP and Aβ in the cells or medium derived from the cell line s12c8 are almost 1.2 fold higher than those from the cell line w5c1 (Figure 5). This observation suggests that the Swedish double mutation in the APP coding sequence enhance the stability of APP and Aβ. 

APP metabolism where the pathway of Aβ formation contains three putative secretases (α, β and γ- secretase) appears to be intact in our cells. To confirm that the secretase pathway was being used in our cells, we modified the activity of the secretases and measured APP, Aβ and Aβ42 levels. We confirmed that both w5c1 and s12c8 have changes in Aβ42 production in response to drug treatments (Figure 6 and 7). Donepezil, which causes an increase in the levels of α-secretase ADAM 10 [25], and galantamin (Gal), which inhibits expression of the β-site APP cleaving enzyme 1 (BACE 1, β-secretase) [26], significantly reducedAβ42 production in both cell lines (Figures 6 and 7). NSAIDs, such as ibuprofen, are known to lower Aβ42 levels [23] by modulating γ-secretase activity [45]. In our cells, ibuprofen, at 250mM, inhibited Aβ42 production (cell content, Figure 6) and secretion (in culture medium, Figure 7), while treatment with aspirin did not show an Aβ42 lowering effect (Figures 6 and 7). Our results suggest that our cells are using the secretase pathway, thus they should be useful for screening of additional drugs that may alter Aβ42 production via this pathway. The inhibitory effects of donepezil, galantamin, and ibuprofen on Aβ42 production showed a consistent difference between the w5c1 and s12c8 cell lines, with the drugs showing stronger effects in the s12c8 cell line (Table 1, Figure 7). The greater sensitivity of the s12c8 cell line, which contains APP with the Swedish double mutation, suggests that it may be most useful for drug screening.

Since our genetically transformed cell lines w5c1 and s12c8 showed detectable Aβ42 production that can be inhibited by known secretase inhibitors and modulators these cells should be useful tool for clarifying mechanisms involved in amyloid metabolism. Our system can easily be manipulated by pharmacological and cell biological procedures, thus studies into the regulation of amyloid production at the levels of transcription, translation, posttranslational modification, and/or degradation, in these cell lines should yield greater insights and lead to therapeutic applications to treat AD.

## Materials and Methods

### 1: Plasmids construct

The complementary pair of zinc finger nucleases (ZFNs) ZFL (pmlm290, plasmid 21872) and ZFR (pmlm292, plasmid 21873) were acquired from Addgene. The ZFNs, ZFL and ZFR, harbor a pair of mutations in the FokI nuclease domain (E490K and I538K, and Q486E and I499L, for ZFL and ZFR, respectively; the “+” and “-“mutation, see 44) that confer heterodimeric behavior to these domains. The backbone vectors were digested with BamHI and XbaI and the zinc finger coding regions were replaced with DNA fragments containing engineered-coding sequences for the left and right hands of the Zinc Finger Proteins (ZFP) that are specific for our target sequences. ZFP mRNAs were designed based on the zinc-finger-framework consensus sequence previously described [46]. ZFPs that recognize each of the specific 3 bp sequences within the chosen target site were designed based on data from the Sigma Advanced Genetic Engineering (SAGE) Labs, and the coding sequences were synthesized by Invitrogen.

Our FokI based, heterodimeric ZFN pair targets an 18bp sequence within the first intron of the *mouse Rosa26 gene*. The sequence is composed of a pair of 9 base sequences (underlined) that flank a 5bp spacer region (shown in bold): 5′- cacctctcc **CGTCA** ggtggtgtt-3′. The two 9bp “cacctctcc” and “ggtggtgtt” DNA sequences are ZFN recognition sites, separated by a 5bp“**CGTCA**” spacer sequence, which would be the ZFN target site where ZFN induced Double Strand Breaks. An 18 bp sequence should be long enough to specify a unique location within mammalian genomes [37–39]. The amino acid sequences of the specificity-determining residues of the three zinc fingers in ZFL are: finger 1, RRHILDR (recognizes GTG); finger 2, RQDNLGR (GAG); and finger 3, QANHLSR (GGA). In ZFR they are: finger 1, AATALRR (GTT); finger 2, EAHHLSR (GGT); and finger 3, IRHHLKR (GGT). 

The ZFN expression constructs contain both a T7 phage promoter and a CMV promoter that allow expression in both prokaryotic and eukaryotic cells. For all expression experiments the ZFN expression plasmids were first linearized by digestion with BstBI and SacI. 

A DNA fragment (0.98kb) corresponding to the ZFN target site (TS) was extracted from genomic DNA of Balb/c 3T3 cells, and cloned into the multiple cloning site of pMD-19T to form the ZFN-TS vector (Figure 1A) which serves as the substrate for in vitro ZFN digestion. 

Components of the Rosa26 targeting vector (pRosa26 wt and pRosa26 swe) (illustrated in Figure S1) are described as follows: The diphtheria toxin (DTA) expression cassette were from plasmid DTA (Addgene plasmid 22730). The homologous recombination arms were extracted from Balb/c 3T3 cells genomic DNA. The Chicken-Actin-CMV promoter (CAG promoter, Addgene plasmid 11150) is a strong ubiquitous promoter, with a CMV enhancer, followed by an expression cassette for green fluorescence protein (GFP) [47]. Immediately after the GFP expression cassette, there is an adenoviral splice acceptor (SA) sequence followed by a loxP site, a neomycin expression cassette, and a strong transcriptional stop sequence (triple SV40 polyadenylation sequence). The transcriptional stop sequence is then followed by another loxP site, in the same orientation as the first, the APP coding sequence, and the bovine growth hormone polyadenylation sequence. APP would only be expressed if the strong transcriptional stop sequence is removed by Cre recombinase mediated recombination between the two loxP sites. A PacI site is located 5' to the SA, and an AscI site is 3' to the bovine growth hormone polyadenytation sites, and were used for digestions win Southern blots. Constructs containing both the wildtype (human APP751 cDNA) and containing the “Swedish” double mutation (human APP751 cDNA with KM670/671NL2) were generated. LV-CRE (addgene plasmid 12105) vector encodes the Cre recombinase with a nuclear location signal (nls) under the control of an internal CMV promoter. To induce Cre expression in cells, we electroporated double digested (KpnI and NotI) LV-CRE, containing the CMV promoter and Cre-nls coding region, into our APP transformed cells.

### 2: *In vitro* characterization of the sequence-specific cleavage by the designed ZFNs

The modified in vitro transcription–translation (IVTT) assay [48] was used to translate the ZFNs to screen their sequence-specific cleavage of DNA. This protocol utilizes phage T7 RNA polymerase to generate mRNAs from our linearized ZFN expression plasmids and the rabbit reticulocyte IVTT system to generate the fusion protein products encoded by these mRNAs in a crude extract to study the sequence-specific cleavage of the ZFN-TS vector. Procedures were in accordance with the manufactures instructions of the TNT® T7 Quick Coupled Transcription/Translation System (Promega).

### 3: Analysis of NHEJ after ZFNs sequence-specific cleavage of in Balb/c 3T3 cells

Balb/c 3T3 cells (ATCC^®^ CCL-164™) were co-transfected with 2.5 ug of linearized (see above) DNA fragments containing the ZFR and ZFL coding region and the CMV promoter. Approximately 1 × 10^6^ cells were transfected with a total of 5 ug of DNA by electroporation at 260v for 30ms following the manufacturer’s protocol. Cells were harvested 48 hours post-transfection, and genomic DNA was isolated using the DNA MasterPure Kit (TaKaRa). ZFN-induced modification of the genomic loci encompassing *Rosa26* was analyzed by PCR amplifying the regions of interest using primer pair primer1 (Table S1). PCR conditions: Initial denaturation 95°C for 10 min, followed by 30 cycles 90°C for 60 s; 60°C for 60 s; 72°C for 30 s. Final elongation was for 10 min at 72°C. 3 ul of the PCR product was assayed on a 1.5% TAE agarose gel to confirm amplification. PCR fragments were T-A cloned into the PMD-19T plasmid vector (TaKaRa). The library of *Rosa26 intron I* segments was transformed into *E. coli* cells and a total of 48 recombinant colonies were sequenced to determine the type and distribution of lesions at the Intron 1 site. Sequences that had >2 bp indel mutations located within the spacer ± 1 bp were considered to be ZFN induced genome modifications. Aligned sequences were further validated by comparison to the mouse genome by BLAST.

### 4: Vectors and cell transduction

To generate targeted insertion of the *Rosa26* targeting sequence linearized ZFN expression plasmids and linearized Rosa26 targeting vector were transfected into cells. Linearized ZFN DNA was as described above. Plasmids pRosa26 (pRosa26wt and pRosa26swe) were linearized at the 5′end of the short homology arm with BstBI. Balb/c 3T3 cells were electroporated with a total of 50ng DNA using a ZFN:donor ratio of 3:1. DNAs were dissolved in electroporation buffer at a working concentration of 15 ng∕μL (pRosa26) together with fragments including both ZFN coding regions and a CMV promoter. Balb/c 3T3 cells, at a concentration of 10^7^/ml (300ul), were electroporated with a mixture of linearized targeting and ZFN expression vectors at 260v for 30ms. Transfected cells were expanded for flow cytometry analysis (FACSCalibur; Becton Dickinson Pharmingen) and genomic DNA extraction. Cells were kept under selection with 600µg/ml G418 for two weeks and a total of 192 single-cell derived clones (96 mutant and 96 wildtype) were obtained by limiting dilution and analyzed for integration into the ZFN-target site by PCR, Southern blot analysis and GFP detection. A total of 7 wildtype and 6 Swedish double mutation APP knock in cell lines were established that had the correct APP insertion. LV-CRE vector was then electroporated into the established cell lines s12 (with the Swedish double mutation APP sequence) and w5 (with wildtype APP sequence) to remove the stop cassette between the two LoxP sites, which allowed the linked of the CAG promoter to the APP cDNA and thus induce APP expression. By limiting dilution, 60 single-cell derived clones (30 mutant and 30 wildtype) were obtained and analyzed for stop cassette removal by PCR and Southern blot analysis. Cell lines generated are named using the following conventions: the first letter w or s refers to wildtype or Swedish double mutation respectively; the number refers of the clone number; c (if present) refers to addition of Cre recombinase; the second number (if present) referees to clone number after Cre addition.

### 5: Preparation of Genomic DNA

Cells were homogenized in lysis buffer (50 mM Tris-HCl, pH 8.0; 100 mM EDTA, pH 8,0; 1% SDS; 100 mM NaCl; 350 μg∕μL Proteinase K) and incubated at 55°C for 2 hours under vigorous shaking. Genomic DNA was isolated after phenol/chloroform extraction and isopropanol precipitation. The pellet was resuspended in 10 mMTris/1 mM EDTA buffer (pH 7.5).

### 6: PCR and Sequence Analysis

Primer pair primer1 was used to amplify the 5′-homology arm/CAG promoter and primer2was used to amplify the junction of the homology arms. To analyze whether the *Rosa26 alleles* had recombined with pRosa26, we used primer3 to amplify the 5′-CAG promoter/Neo coding sequence. Primer4was used to amplify the junction of the two LoxP sites (Figure 2A) to analyze stop cassette excision from the *Rosa26* alleles in the activated APP expression cell lines. Amplification was performed using PrimerSTAR HS DNA Polymerase (TaKaRa), which minimizes the amount of nonspecific amplification in 20-μl reactions. PCR used the following cycling conditions: 1 cycle at 95°C for 5 min, 38 cycles of [60 s at 94°C; 60 s at 60°C; 3min at 72°C], 1 cycle at 72°C for 10min. PCR products from cells exhibiting homologous recombination events were directly sequenced and compared to the pRosa26 vector sequence. 

### 7: Southern Blot Analyses

Digested genomic DNAs were separated on 1% agarose gels and blotted on to Hybond Np membranes (GE Healthcare). Membranes were then UV-cross-linked and preincubated in Easy hybridize buffer (Roche) for 10 h at 42°C under rotation. The probes were isolated as 204-bp (sp) and 472bp (spn) fragments. The probe sp (204bp) (located upstream of the ZFN target site right after the XbaI site as shown in Figure 2A) was prepared by PCR using the primers 5' GGCTAACCTGGTGTGTGG 3' and 5' AATACTCCGAGGCGGATC 3' using the full length homologous recombination arm plasmid as template with a PCR DIG synthesis kit (Roche). The probe spn (472bp) (located in the APP coding region as shown in Figure 2A) was prepared by PCR using primers 5' AGAGAGGCTTGAGGCCAA 3' and 5' AGGCACGTTGTAGAGCAG 3',and the full-length hAPP cDNA plasmid clone as template, using a PCR DIG synthesis kit (Roche) (see Figure S2). Hybridization probes were labeled using the *DIG High Prime DNA Labeling and detection starter kit* (Roche) and hybridized to membranes overnight at 42°C. Membranes were washed and hybridized probe was detected immunologically.

### 8: Immunofluorescence

Cells (confluence 80%) were washed three times in phosphate buffered saline (PBS, pH 7.2), and then fixed in 4% Paraformaldehyde in PBS containing 0.1% (vol/vol) Triton X-100 (PBST) at room temperature for 10 min. Prior to immunofluorescence staining, nonspecific binding sites were blocked by incubating the cells in 5% Bovine Serum Albumin (BSA, Boehringer) in PBST for 30 min at 37°C. Primary antibodies for APP (CST, #2452) and Actin (CST, #4967) were used. Antibodies were diluted in PBST, and incubation was carried out overnight at 4°C. Cells were washed three times with PBST. Incubation with secondary antibodies [goat anti-Rabbit antibody conjugated with tetramethylrhodamineisothiocyanate (*TRITC*) (Molecular Probes Inc.)] diluted in blocking buffer was carried out for 30 min at 37°C. Cells were washed three times in PBST. 

### 9: Western Blot Analyses

Western blot analysis was performed as previously described with minor modification [49]. Cell lysates were obtained from cells that were extensively washed with PBS, and lysed directly in three decontamination cell lysate solutions. Protein concentrations were determined using ultraviolet spectrophotometer. Equal amounts (100 μg/lane) of cell lysates or culture medium were separated by sodium dodecyl sulfate polyacrylamide gel electrophoresis (SDS-PAGE) or Tricine–SDS-PAGE and transferred to polyvinylidenedifluoride membranes (Hybond-P; GE Healthcare). The blots were probed with an appropriate primary antibody, followed by HRP-conjugated anti-rabbit IgG (Cell Signaling Technology, CST). Protein bands were visualized using an enhanced chemiluminescence (ECL) detection method (Bio-Rad), and band intensity was analyzed with a densitometer (LAS-4000; GE Healthcare). Immunoreactive protein content in each sample was calculated based on a standard curve constructed using Aβ42; loading quality of the samples was assessed based on a standard curve constructed using BSA. Each set of experiments was repeated at least 3 times to confirm the results. The level of β-actin protein, measured by quantitative Western blotting using β-actin antibody (CST, #4967), was used as an extraction and loading control.

### 10: Activity of β-secretase inhibitor, α-secretase activator and NSAIDs

Cells were incubated in 96 well plates for High content and 6 well plates for Western blot analysis at a density of 2.0×104 /ml cells per well overnight. Ibuprofen, aspirin, donepezil, galantamin and chlorpromazine were prepared in PBS and drugs were added to cells in fresh medium at final concentrations of 250 mM, 250 mM, 1.5μM, 0.9μM and 250 mM, respectively. Treatment was continued for 24 hours. Cells in 96 well plates were prepared for High Content to examine changes in APP and Aβ42 expression in cells. Cells in 6 well plates were prepared for Western Blots to analyze changes in peptide concentrations in both cells and in cell culture medium. All experiments were repeated 3 times and results were from either a representative experiment or all experiments (mean ± SD) are shown. 

### 11: Statistical Analysis

All data are expressed as mean ± SD. Comparisons of the means among more than 3 groups were done by one-way or two-way ANOVA, followed by *post-hoc* tests (PRISM, GraphPad software). P values ≤0.05 indicate significant differences.

## Supporting Information

Figure S1 **Map of the targeting vectors (pRosawt and pRosaswe).** pRosa26swe vector contains the human APP751 cDNA with the “Swedish” double mutation (KM670/671NL2). pRosa26wt vector contains the wildtype APP751 cDNA. Transcription is driven by the chicken gamma-actin promoter (CAG promoter, with CMV enhancer, followed by an expression cassette for Green Fluorescence Protein (GFP)). APP coding region (APPwt and APPswe) is downstream of the adenovirus splice acceptor sequence followed by a LoxP site, PGK-Neo-pA and three copies of the SV40 polyA signal, which function as a stop cassette, a second LoxP site, in the same orientation as the first, with all of these being upstream of the bovine growth hormone polyadenylation sequence. Homologous recombination arms are located on the two sides of these fragments. DTA works as a selective marker.
(TIF)

Figure S2 **Southern blot analysis of transgenic APP expression cell lines.** The probe sp (204bp) was prepared using the full-length short arm plasmid clone as template with primers 5' GGCTAACCTGGTGTGTGG 3' and 5' AATACTCCGAGGCGGATC 3' and a PCR DIG synthesis kit (Roche). The probe spn (472bp) was prepared using the full-length wildtype hAPP cDNA plasmid clone as template with primers 5' AGAGAGGCTTGAGGCCAA 3' and 5' AGGCACGTTGTAGAGCAG 3' and a PCR DIG synthesis kit (Roche). Locations of these two probes are shown in Figure 2A. Genomic DNA (6 μg) was digested overnight with 30 units of the restriction enzyme XbaI (for initially transfected APP expression cell lines) and SacII (for cell lines treated with Cre receobinase (LV-CRE)) in a volume of 30 μL. (A) Identification of cells containing the APP expression construct. Southern blot analysis using probe sp was conducted to confirm the insertion of the APP fragment into the genomic loci of *Rosa26*. Genomic DNA was digested with restriction endonuclease *Xba*I. The wild-type band (wt) has a calculated size of 4.3 kb. A recombination event in one of the two chromosomes yields bands corresponding to lengths of 2.3 kb and 4.3 kb. Cells with recombination events in both chromosomes have bands that correspond to a length of 2.3 kb. Control (lane1) was genomic DNA of Balb/c 3T3 cells. Lane 3 is named w5, a cell line that has the hAPPwt knocked-in in both chromosomes. Lanes 5, 6 and 7 are named s7, s9 and s12 are hAPPswe knock-in cell lines that harbor recombination events in both chromosomes. (B) Identification of cell lines with activated APP expression. Southern blot analysis was conducted using probe spn to confirm the deletion of the stop cassette between two LoxP sites. Genomic DNA was digested with the restriction endonuclease SacII. Before deletion, the size of the band is calculated to be 5.8 kb. Recombination between LoxP sites in one of the two chromosomes will yields bands of lengths 3.1 kb and 5.8 kb. Cells with the stop cassette deleted in both chromosomes will have a band of 3.1 kb. Control (lane1 and 2) was genomic DNA of w5 and s12. Lanes 4, 5, 6 and 7 are w5c2, w5c3, w5c5 and s12c8, cell lines that have the stop cassette deleted in one of the two chromosomes. Lane3 is named w5c1, a cell line that harbors the stop cassette deletion in both chromosomes.
(TIF)

Table S1 **PCR primers used in characterizing cells.**
(DOCX)
