# Supplementary figures and images for: An Over Expression APP Model for Anti-Alzheimer Disease Drug Screening Created by Zinc Finger Nuclease Technology
Source: PLoS One. 2013 Nov 6;8(11):e75493. doi: 10.1371/journal.pone.0075493 (PMC3819351; doi:10.1371/journal.pone.0075493)

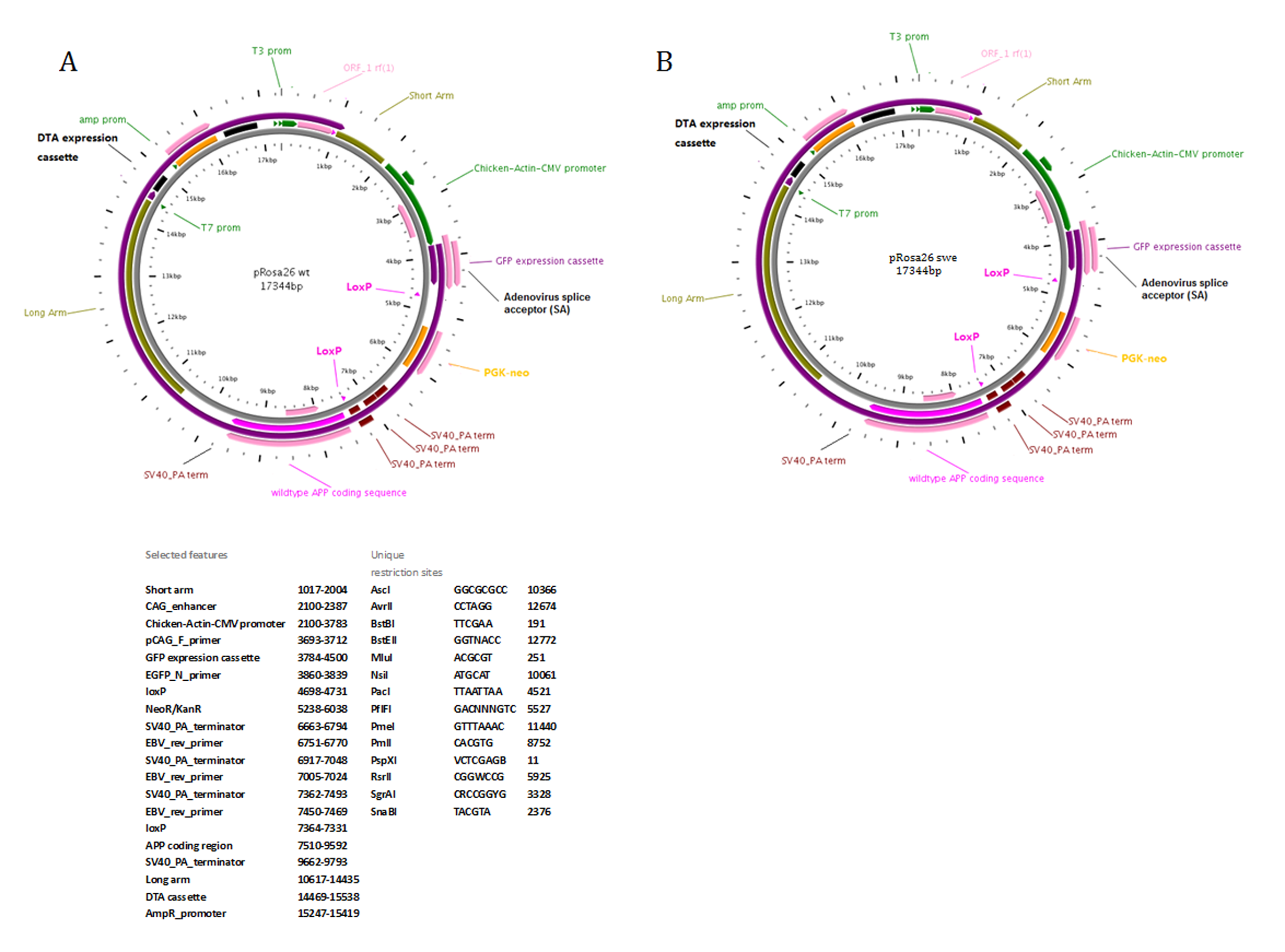

Supplement: Figure S1 — Map of the targeting vectors (pRosawt and pRosaswe). pRosa26swe vector contains the human APP751 cDNA with the “Swedish” double mutation (KM670/671NL2). pRosa26wt vector contains the wildtype APP751 cDNA. Transcription is driven by the chicken gamma-actin promoter (CAG promoter, with CMV enhancer, followed by an expression cassette for Green Fluorescence Protein (GFP)). APP coding region (APPwt and APPswe) is downstream of the adenovirus splice acceptor sequence followed by a LoxP site, PGK-Neo-pA and three copies of the SV40 polyA signal, which function as a stop cassette, a second LoxP site, in the same orientation as the first, with all of these being upstream of the bovine growth hormone polyadenylation sequence. Homologous recombination arms are located on the two sides of these fragments. DTA works as a selective marker. (TIF) [file pone.0075493.s001.tif]

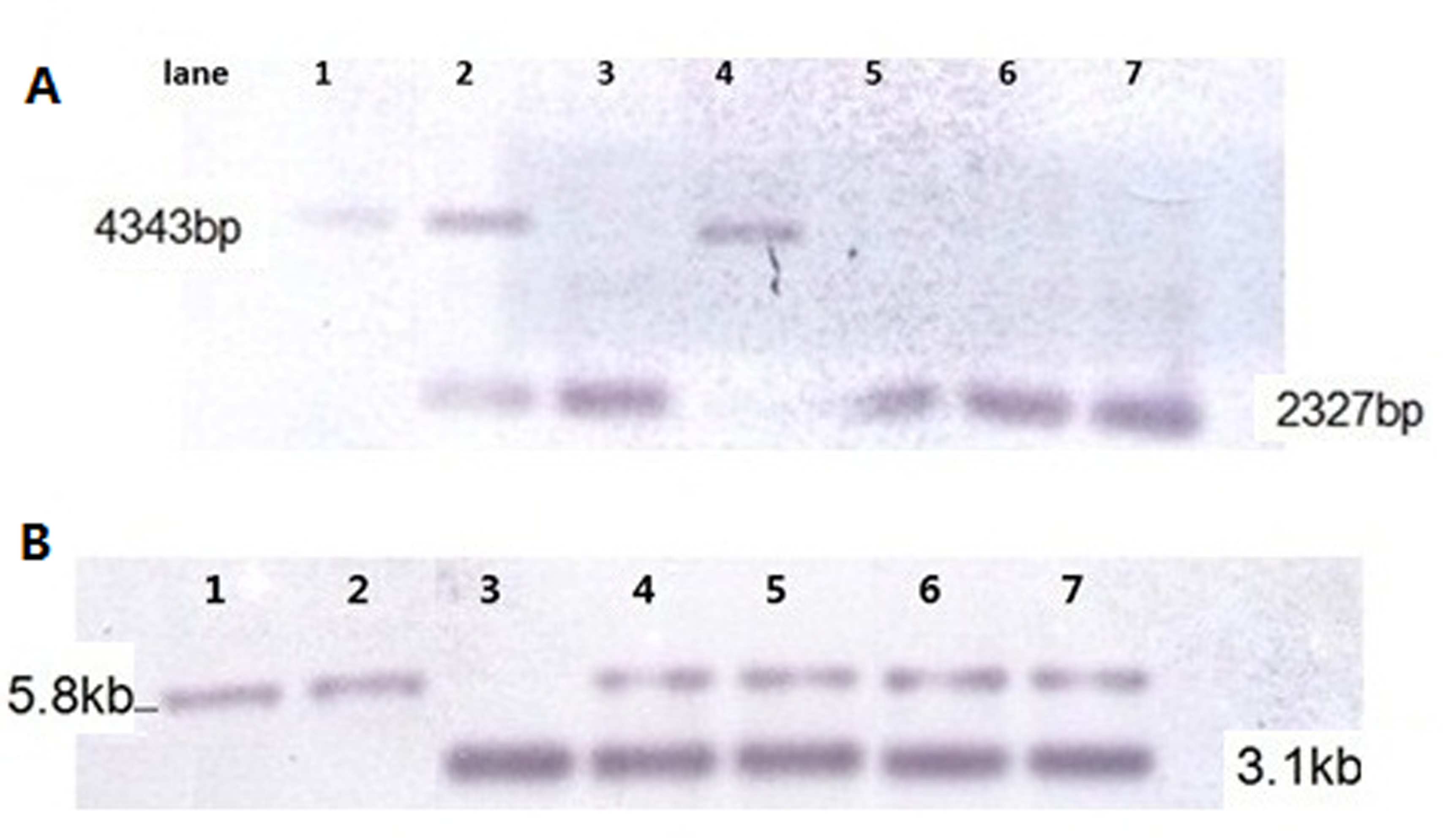

Supplement: Figure S2 — Southern blot analysis of transgenic APP expression cell lines. The probe sp (204bp) was prepared using the full-length short arm plasmid clone as template with primers 5' GGCTAACCTGGTGTGTGG 3' and 5' AATACTCCGAGGCGGATC 3' and a PCR DIG synthesis kit (Roche). The probe spn (472bp) was prepared using the full-length wildtype hAPP cDNA plasmid clone as template with primers 5' AGAGAGGCTTGAGGCCAA 3' and 5' AGGCACGTTGTAGAGCAG 3' and a PCR DIG synthesis kit (Roche). Locations of these two probes are shown in Figure 2A. Genomic DNA (6 μg) was digested overnight with 30 units of the restriction enzyme XbaI (for initially transfected APP expression cell lines) and SacII (for cell lines treated with Cre receobinase (LV-CRE)) in a volume of 30 μL. (A) Identification of cells containing the APP expression construct. Southern blot analysis using probe sp was conducted to confirm the insertion of the APP fragment into the genomic loci of Rosa26. Genomic DNA was digested with restriction endonuclease XbaI. The wild-type band (wt) has a calculated size of 4.3 kb. A recombination event in one of the two chromosomes yields bands corresponding to lengths of 2.3 kb and 4.3 kb. Cells with recombination events in both chromosomes have bands that correspond to a length of 2.3 kb. Control (lane1) was genomic DNA of Balb/c 3T3 cells. Lane 3 is named w5, a cell line that has the hAPPwt knocked-in in both chromosomes. Lanes 5, 6 and 7 are named s7, s9 and s12 are hAPPswe knock-in cell lines that harbor recombination events in both chromosomes. (B) Identification of cell lines with activated APP expression. Southern blot analysis was conducted using probe spn to confirm the deletion of the stop cassette between two LoxP sites. Genomic DNA was digested with the restriction endonuclease SacII. Before deletion, the size of the band is calculated to be 5.8 kb. Recombination between LoxP sites in one of the two chromosomes will yields bands of lengths 3.1 kb and 5.8 kb. Cells with the stop cassett [file pone.0075493.s002.tif]
