# Supplementary material for: An Over Expression APP Model for Anti-Alzheimer Disease Drug Screening Created by Zinc Finger Nuclease Technology
Source: PLoS One. 2013 Nov 6;8(11):e75493. doi: 10.1371/journal.pone.0075493 (PMC3819351; doi:10.1371/journal.pone.0075493)
Supplement: Table S1 — PCR primers used in characterizing cells. (DOCX) [file pone.0075493.s003.docx]

**Table S1: PCR primers used in characterizing cells**

| Primer pair | Sense primer | Anti-sense primer | Product size (bp) |
| --- | --- | --- | --- |
| primer1 | 5' ACCAGACAGATTAGTTAC 3' | 5' TTGGATTCTCAATACATC 3' | 204 |
| primer2 | 5' ACCAGACAGATTAGTTAC 3' | 5' TACCGTAAGTTATGTAACGCG 3' | 215 |
| primer3 | 5'GCTTAATTAATGGGATCGGCCATTGA 3‘ | 5'TACCCGGGATAAGAACTCGTCAAGAAG 3‘ | 801 |
| primer4 | 5' AACGAGAAGCGCGATCACA 3' | 5' GGATCCAGACATGATAAGA 3' | 216 or 2516 |
